# Membrane-Free Stem Cell Extract Enhances Blood–Brain Barrier Integrity by Suppressing NF-κB-Mediated Activation of NLRP3 Inflammasome in Mice with Ischemic Stroke

**DOI:** 10.3390/life12040503

**Published:** 2022-03-29

**Authors:** Ji Hyeon Ryu, Yeonye Kim, Min Jae Kim, Jisu Park, Ji Won Kim, Hye Sook Park, Young Sil Kim, Hwa Kyoung Shin, Yong-Il Shin

**Affiliations:** 1 Research Institute for Convergence of Biomedical Science and Technology, Pusan National University Yangsan Hospital, Yangsan 50612, Gyeongnam, Korea; wlgus9217@naver.com (J.H.R.); yeonyekim@naver.com (Y.K.); jiss5022@naver.com (J.P.); hahahakiki84@naver.com (J.W.K.); 2 Department of Korean Medical Science, School of Korean Medicine, Pusan National University, Yangsan 50612, Gyeongnam, Korea; dpxls0@pusan.ac.kr (M.J.K.); julie@pusan.ac.kr (H.K.S.); 3 T-Stem Co., Ltd., Changwon 51573, Gyeongnam, Korea; ceo@t-stem.com (H.S.P.); askand@naver.com (Y.S.K.); 4 Department of Rehabilitation Medicine, School of Medicine, Pusan National University, Yangsan 50612, Gyeongnam, Korea

**Keywords:** membrane-free stem cell extract, ischemic stroke, blood–brain barrier, brain edema, matrix metalloproteinase, Toll-like receptor 4, NF-kappa B

## Abstract

Membrane-free stem cell extract (MFSCE) of human adipose tissues possesses various biological activities. However, the effects of MFSCE on blood–brain barrier dysfunction and brain damage are unknown. In this study, we determined the role of MFSCE in an ischemic stroke mouse model. Mice were treated with MFSCE once daily for 4 days and 1 h before ischemic damage. Experimental ischemia was induced by photothrombosis. Pretreatment with MFSCE reduced infarct volume and edema and improved neurological, as well as motor functions. Evans blue leakage and water content in the brain tissue were reduced by MFSCE pretreatment relative to those in the vehicle group. MFSCE increased the expression of the tight junction proteins zonula occludens 1 and claudin-5, as well as vascular endothelial-cadherin, but decreased that of matrix metalloproteinase 9. Notably, MFSCE treatment decreased cell death and the level of NOD-like receptor protein 3 inflammasome, consistent with the downregulated expression of the pro-inflammatory cytokines interleukin (IL)-1β and IL-18 in the ischemic brain. These effects might have occurred via the suppression of the expression of Toll-like receptor 4 and activation of nuclear factor-κB. The results highlighted the potential of MFSCE treatment as a novel and preventive strategy for patients at a high risk of ischemic stroke.

## 1. Introduction

Stroke is the second leading cause of death and is associated with high incidence, mortality, and disability rates worldwide [1]. Stroke induces irreversible damage to the ischemic core and potentially reversible penumbra in the surrounding tissue [2]. The principal aim of therapy for acute ischemic stroke is the prompt rescue of penumbra, which requires the delivery of sufficient drugs to the ischemic brain regions [3]. Despite several clinical trials on the treatment of stroke, recombinant tissue plasminogen activator (rtPA) is the only drug that has been approved by the Food and Drug Administration for the treatment of acute ischemic stroke [4]; this drug dissolves clots, thereby restoring blood flow [5]. Unfortunately, to date, only a few patients have received effective treatment with rtPA, owing to the narrow time window and safety problems associated with increased intracerebral hemorrhage and toxicity [5].

Blood–brain barrier (BBB) dysfunction by stroke is the key factor limiting the treatment time window of rtPA [6]. In addition, rtPA might cause injury to the BBB via the activation of matrix metalloproteinases (MMPs) [7]. Upregulation of MMPs plays an important role in thrombolysis-mediated BBB leakage and edema, leading to intracranial bleeding [8]. Among all MMP subtypes, MMP-2 and MMP-9 have been the most investigated in ischemic stroke, with MMP-9 being reported as the principal culprit in the disruption of BBB and neuronal damage [9]. Aberrant MMP-9 degrades tight junction and basal membrane proteins [10], as a result of increased infiltration of inflammatory mediators, which can cause cerebral edema, hinder nutrient exchange, and induce neurotoxicity, thereby exacerbating ischemic stroke [11]. Thus, novel therapeutic targets to preserve the integrity of BBB after ischemic brain injury should be identified.

Neuronal death by stroke is mediated by a macromolecular complex termed the inflammasome. The nucleotide-binding oligomerization domain (NOD)-like receptor (NLR) pyrin domain-containing (NLRP) inflammasome is composed of the NLRP1/3 receptor, apoptosis-associated speck-like protein containing a caspase recruitment domain (ASC), precursor caspase-1, precursor caspase-11, or X-linked inhibitor of apoptosis (XIAP) or both [12]. The activated NLRP inflammasome converts precursor caspase-1 into cleaved caspase-1 via proximity-induced autoactivation [13]. The activation of caspase 1 leads to the maturation of pro-inflammatory cytokines, such as interleukin (IL)-1β and IL-18, which are released into the extracellular environment [13]. Increased levels of NLRP inflammasomes, IL-1β, and IL-18 might be induced under ischemic conditions by the activation of Toll-like receptors (TLRs) and the NF-κB signaling pathway [14]. Thus, therapeutic interventions that target the activation of the inflammasome might provide new opportunities for the treatment of stroke.

Adipose-derived stem cells (ADSCs) have garnered considerable interest because of their potential therapeutic applications in regenerative medicine. In particular, ADSCs can be easily isolated from abundant adipose tissues with minimal invasiveness, are adherent to culture flasks, can be expanded in vitro, and have the capacity to differentiate into multiple cell lineages [15]. Interestingly, ADSCs have been reported to lead to the functional recovery of cerebral lesions and reduce apoptosis in experimental ischemic stroke [16]. In addition, ADSCs can suppress inflammatory factors, ameliorate neurological disorders, and reduce brain infarction volume [17]. Recently, membrane-free stem cell extract (MFSCE), in which the membrane of human adipose tissue-derived stem cells is removed using patented technology, has been developed to surmount the limitations of cell-mediated immune rejection and the high cost of stem cell therapy [18]. Furthermore, MFSCE contains several bioactive molecules that have various bioactivities [18] and exerts several beneficial health effects. However, the protective effect of MFSCE on ischemic brain injury remains unknown. Here, we investigated the therapeutic potential and mechanisms involved in MFSCE-mediated disruption of the BBB and brain damage elicited by focal cerebral ischemia, to elucidate the clinical applications of MFSCE.

## 2. Materials and Methods

### 2.1. Preparation of MFSCE

MFSCE was provided by T-Stem Co. (Changwon, Korea) and was prepared as previously described [18]. Briefly, human adipose tissues were separated and purified, and the extracted cells were cultured in a serum-free medium (PowerStem MSC1; PAN-Biotech, Aidenbach, Germany) at 37 °C under 5% CO_2_. The donor of human adipose tissues was a healthy female in her 20s with 2-degree obesity (BMI 25–29.9). The adipose tissues were obtained after blood tests, which indicated the suitability of the obtained tissues. Cells were subcultured until passages 6 to 8 after reaching 70–80% confluence. The cells were then collected and ultrasonicated to remove the membranes. The cell debris was removed by centrifugation at 800–1500*×* *g*, following successive filtrations. The final MFSCE product was determined to be non-toxic based on nine safety tests, performed by the Good Laboratory Practice accreditation authority (Chemon Inc., Yongin, Korea). 

### 2.2. Mouse Model of Focal Cerebral Ischemia and Treatment with MFSCE

**Figure 1:**
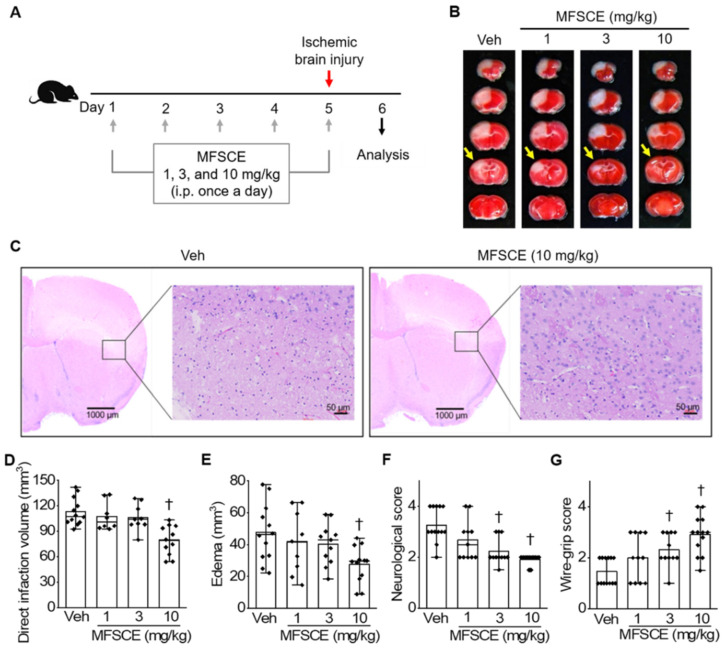
Pretreatment of membrane-free stem cell extract (MFSCE) reduced brain damage and improved functional behavior after ischemic brain injury (*n* = 8–12). (**A**) Scheme of the experimental protocol. Mice were administered a daily intraperitoneal (i.p.) injection of MFSCE (1, 3, and 10 mg/kg) in saline for 4 days and 1 h before the induction of ischemic stroke. Focal cerebral ischemia was induced by photothrombosis of cortical microvessels. (**B**) Representative images of coronal brain sections stained with TTC in vehicle (Veh) and MFSCE-treated mice. (**C**) Representative images of coronal brain sections stained with hematoxylin and eosin (H&E)-stained cerebral cortex after ischemic injury. (**D**) Direct infarct volume and (**E**) edema. (**F**) Neurological score and (**G**) wire-grip score. ^†^ *p* < 0.05, compared to Veh.

Male C57BL/6 mice aged 5 weeks were obtained from Orient Bio (Seongnam, Korea) and housed in a pathogen-free containment facility. All experiments were approved by the Institutional Animal Care and Use Committee of the Pusan National University Yangsan Hospital in accordance with the National Institute of Health Guidelines [2021-029-A1C0(0)]. The induction of ischemia and treatment protocols used in this study were as described previously [19]. Focal cerebral ischemia was induced by photothrombosis of cortical microvessels. Briefly, the mice were anesthetized with isoflurane (Hana Pharm Co., Ltd., Hwaseong, Korea; 2% induction and 1.5% maintenance, in 80% N_2_O and 20% O_2_) and intraperitoneally injected with 0.1 mL of 10 mg/mL Rose Bengal (Sigma-Aldrich, St. Louis, MO, USA) in saline 5 min before illumination. Each mouse was fixed on a stereotaxic frame (David Kopf Instruments, Tujunga, CA, USA), with the skull exposed. A fiber-optic bundle containing a CL 6000 LED cold light source (Carl Zeiss, Jena, Germany) was positioned on the sensorimotor cortex of the exposed skull (2.4 mm lateral from the bregma) and illuminated for 15 min. After illumination, the scalp was sutured, during which the mice were allowed to recover under a heating lamp and returned to their home cages. The body temperature of the mice was maintained at 37.5 °C during surgery using a heating pad (Harvard Apparatus, Holliston, MA, USA). As shown in Figure 1A, the mice were intraperitoneally administered MFSCE (1, 3, and 10 mg/kg) in saline once a day for 4 days and 1 h before the induction of ischemia. For measuring the effect of post-treatment with MFSCE, mice were administered a daily intraperitoneal injection of MFSCE in saline twice a day every 12 h for three days from 2 h after the induction of ischemic stroke (Supplementary Materials Figure S1).

### 2.3. Infarct Volume and Edema

The mice were sacrificed 24 h after ischemic injury, and their brains were immediately removed. Subsequently, 2-mm-thick sections were cut from the cerebral infarct part of the brain and stained with 2,3,5-triphenyltetrazolium chloride (TTC, Sigma-Aldrich). The infarct size was analyzed using ImageJ software (version 1.49, National Institutes of Health, Bethesda, MD, USA). The ipsilateral area with direct damage was included in the direct infarct volume, which was calculated using the following formula: contralateral hemisphere (mm^3^) – undamaged ipsilateral hemisphere (mm^3^). The edema was calculated by subtracting the direct from the indirect infarct volume [20].

### 2.4. Neurological Deficit Score

Neurological deficits in each mouse were evaluated 24 h after cerebral ischemic injury using the neurological scoring system: 1 = turning to the ipsilateral (uninjured) side when grasped by the tail, 2 = turning to the opposite side and difficulty in bearing weight, 3 = inability to support weight in the opposite direction, 4 = no spontaneous movement [21].

### 2.5. Wire-Grip Test

The motor function of mice was measured 24 h after ischemic injury using the following scoring system: 1 = inability to grasp the wire; 2 = grasping the wire using both forelimbs and hind paws, but not the tail; 3 = grasping the wire using both fore and hind paws as well as the tail, without movement; 4 = moving on the wire using both fore paws, hind paws, and tail; 5 = moving freely on the wire [21].

### 2.6. Evans Blue Extravasation and Water Content

The integrity of BBB was evaluated using Evans blue extravasation. Briefly, Evans blue (Sigma-Aldrich; 2% in saline, 4 mL/kg) was intravenously administered at the onset of ischemia. The mice were euthanized and then transcardially perfused with phosphate-buffered saline (PBS; Welgene, Gyeongsan, Korea) to remove the intravascular dye 24 h after cerebral ischemia. Next, each hemisphere was weighed, homogenized in 2 mL of N,N-dimethylformamide (Sigma-Aldrich), incubated for 24 h at 55 °C, and then centrifuged at 16,600*×* *g* for 20 min. The absorbance of the supernatant at 620 nm was subsequently measured by spectrophotometry, and the results are expressed as μg/g tissue calculated using a standard curve [22]. The water content in the brain tissue was also measured using the wet and dry weight method at 24 h after cerebral ischemia. More specifically, the brain hemispheres were weighed before and after drying at 100 °C for 48 h, and the percentage of water content was calculated as 100 × (wet weight–dry weight)/wet weight [23]. 

### 2.7. Real-Time Polymerase Chain Reaction

The total RNA was isolated from the ischemic cortex using the TRIzol Reagent (Invitrogen, Carlsbad, CA, USA), and cDNA was synthesized using the amfiRivert Platinum cDNA synthesis master mix (GenDEPOT, Barker, TX, USA) according to the manufacturer’s instructions. Real-time polymerase chain reaction (PCR) was performed to quantify the level of MMP-2 and MMP-9 mRNAs in the ischemic cortex using a Rotor-Gene Q real-time PCR system (Qiagen, Hilden, Germany) with a FastStart Essential DNA Green Master (Roche Diagnostics, Mannheim, Germany). The following primer sequences were used: *MMP-2*, forward: 5′-AGATCTTCTTCTTCAAGGACCGGTT-3′; reverse: 5′-GGCTCCTCAGTGGCTTGGGGTA-3′; *MMP-9*, forward: 5′-TGAATCAGCTGGCTTTTGTG-3′; reverse: 5′- ACCTTCCAGTAGGGGCAADT-3′. The relative abundance of mRNA was calculated after normalization to glyceraldehyde-3-phosphate dehydrogenase (*GAPDH*) mRNA.

### 2.8. Western Blotting

Protein lysates (*n* = 3 per group) were obtained from ischemic cortices using the RIPA buffer (Thermo Fisher Scientific, Rockford, IL, USA) containing a protease inhibitor cocktail (GenDEPOT). The lysates were separated by 8%–15% sodium dodecyl sulfate-polyacrylamide gel electrophoresis (SDS-PAGE), and the proteins were transferred on to polyvinylidene difluoride (PVDF) membranes (Millipore, Darmstadt, Germany). The membranes were blocked with 5% skimmed milk and incubated with specific primary antibodies at 4 °C overnight. The primary antibodies used were as follows: zonula occludens 1 (ZO-1; 1:1000; Invitrogen, Carlsbad, CA, USA), claudin-5 (1:1000; Invitrogen), vascular endothelial-cadherin (VE-cadherin; 1:1000; Invitrogen), MMP-2 (1:1000; Abcam, Cambridge, MA, USA), MMP-9 (1:1000; Abcam), NLRP1 (1:1000; Novus Biologicals, Littleton, CO, USA), NLRP3 (1:1000; Novus Biologicals), ASC (1:1000; Santa Cruz Biotechnology, Dallas, TX, USA), caspase-1 (1:1000; Novus Biologicals), caspase-11 (1:1000; Novus Biologicals), IL-1β (Cell Signaling, Danvers, MA, USA), IL-18 (1:1000; Abcam), TLR-2 (Bioworld Technology, Louis Park, MN, USA), TLR-4 (1:1000; Bioworld Technology), myeloid differentiation factor88 (MyD88, 1:1000; Cell Signaling), NF-κB (1:1000; Cell Signaling), Lamin B1 (1:1000; Cell Signaling), β-tubulin (1:1000; Cell Signaling), and β-actin (1:3000; Sigma). The membranes were then washed three times with Tris-buffered saline with Tween-20 (TBST) and incubated with secondary antibodies conjugated with horseradish peroxidase. Immunodetection was performed using an enhanced chemiluminescence kit (Amersham Pharmacia, Piscataway, NJ, USA) with Fusion Solo X (Vilber Lourmat, Collegien, France). Each band was quantitatively determined using ImageJ software (NIH).

### 2.9. Histological Examination

The mice were perfused with cold PBS followed by 4% paraformaldehyde (Fujifilm Wako Pure Chemical Corporation, Osaka, Japan) 24 h after focal cerebral ischemia induction. Immediately thereafter, the tissues were harvested and fixed for 24 h in the same fixative solution and incubated overnight at 4 °C. The tissues were embedded in paraffin and cut into 5 µm-thick sections. For hematoxylin and eosin (H&E) staining, paraffin was removed from the tissue sections using xylene, and the tissue sections were rehydrated using a graded ethanol series (100% to 70% ethanol). The tissue sections were then washed with tap water for 5 min, and section slides were immersed in hematoxylin solution for 5 min. After checking the degree of hematoxylin staining, eosin staining was performed for 1 min. The sections were dehydrated through a graded ethanol series (70% to 100% ethanol, each 3 min), removed from xylene, and mounted using mounting medium (Fisher Chemical, Geel, Belgium).

The terminal deoxynucleotidyl transferase dUTP nick end labeling (TUNEL) assay was conducted using the DeadEndTM Fluorometric TUNEL System kit (Promega Corporation, Madison, WI, USA). For 4′,6-diamidino-2-phenylindole (DAPI) staining, brain sections were incubated with DAPI (5 μg/mL). Images of TUNEL-positive cells in the brain tissues were obtained using a fluorescence microscope (Axio Imager M1, Carl Zeiss).

### 2.10. In Vivo Safety Evaluation

Healthy 6-week-old male C57BL/6 mice were randomly divided into two groups (n = 7–8). Each group received a single dose of an i.p. injection of MFSCE (10 mg/kg) or saline (control) per day for 5 days. The body weights of the mice were measured daily. Blood and tissue samples were collected 24 h after the last administration for hematologic and histochemical analyses. The levels of serum aspartate transaminase (AST) and alanine transaminase (ALT) were measured by GC Labs (Yongin, Korea), which uses the International Federation of Clinical Chemistry standard method. ALT and AST levels were analyzed by measuring enzymatic activity through a colorimetric method without using pyridoxal phosphate. In a test tube, 1 mL of substrate solution (AST, 2 mmol/L α-ketoglutarate and 200 mmol/L aspartate; ALT, 2 mmol/L α-ketoglutarate and 200 mmol/L alanine) and 0.2 mL of serum were added and incubated at 37 °C for 30 min (ALT) or 1 h (AST). Immediately after the completion of the reaction, 1 mL of a coloring solution (2,4-dinitrophenyl hydrazine, 1 mmol/L) was added, followed by incubation at room temperature for 20 min, and then 10 mL of 0.4 N sodium hydroxide was added to measure the absorbance at 520 nm (Modular Analytics, Roche, Germany). Distilled water was used as a blank. The lung, heart, liver, kidney, spleen, thymus, and testis tissues were fixed with 4% paraformaldehyde for 24 h and embedded in paraffin. Each sample was cut into 5 μm-thick sections, processed for routine H&E staining, and then visualized using a virtual microscope (Axio Scan.Z1; Carl Zeiss). 

### 2.11. Statistical Analysis

Significant differences between groups were analyzed using an unpaired Student’s *t-*test or one-way analysis of variance with post hoc comparisons using Bonferroni test (OriginPro2020, OriginLab Corp., Northampton, MA, USA). *p* < 0.05 was considered as the criterion for statistical significance. 

## 3. Results

### 3.1. Pretreatment with MFSCE Improved Stroke Outcomes in Mice with Ischemic Stroke

We used a photothrombotic stroke mouse model to investigate whether pretreatment with MFSCE could improve functional outcomes in acute ischemic brain injury. We observed the presence of white infarcted brain tissue in 2,3,5-triphenyltetrazolium chloride (TTC)-stained brain sections, whereas normal regions of the brain tissue appeared red. We also observed an extensive infarct area in the vehicle group, whereas pretreatment with MFSCE at a dose of 10 mg/kg reduced this area (Figure 1B). We performed H&E staining to observe histopathological changes in the neurons of brains in the vehicle and MFSCE-treated groups (Figure 1C). A large number of neurons in the vehicle group were shrunken, swollen, and showed karyopyknosis. However, we noticed that the extent of damage was alleviated in the group pretreated with 10 mg/kg MFSCE, and the number of normal neurons was increased compared with that in the vehicle group. Moreover, we found that both direct infarct volume and edema were considerably decreased in the mice treated with MFSCE at a dose of 10 mg/kg, but not in those treated with 1 or 3 mg/kg MFSCE. The result showed insignificant changes in the infarct volume and edema compared with those in the vehicle group (*p* < 0.05; Figure 1D,E). We detected that the neurological deficits (Figure 1F) and motor dysfunction (Figure 1G) by ischemia were considerably improved in the 3 and 10 mg/kg MFSCE-treated groups than in the vehicle group (*p* < 0.05). Therefore, we used a high concentration of MFSCE (10 mg/kg) in the subsequent experiments. 

We investigated whether post-treatment with MFSCE improves functional recovery after cerebral ischemia. Our results suggested that post-treatment with MFSCE did not reduce the cerebral ischemic size or amend neurological signs after cerebral ischemia (Figure S1).

### 3.2. MFSCE Prevented BBB Destruction and Brain Edema by Ischemia

**Figure 2:**
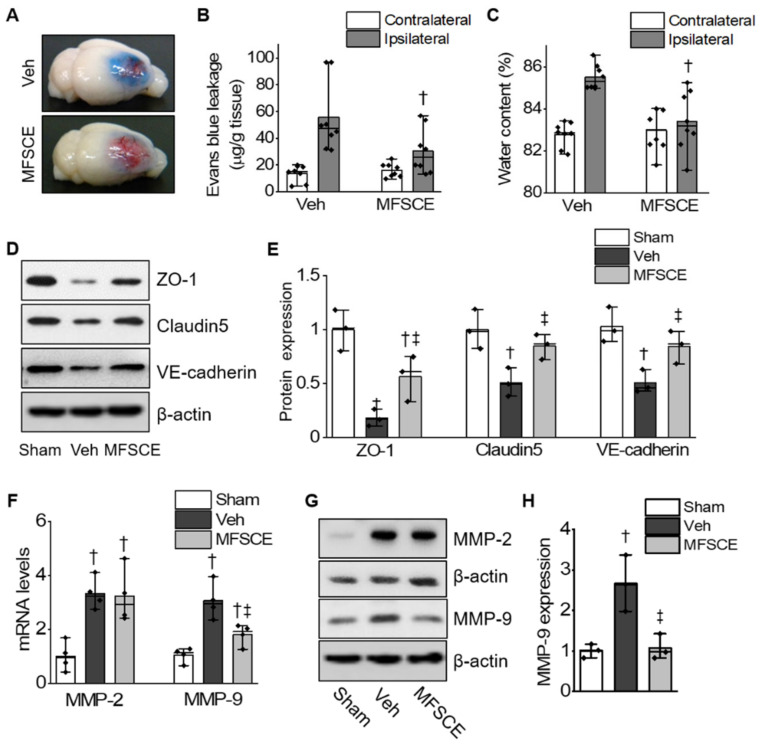
MFSCE increased tight junction proteins and reduced cerebral edema. (**A**) Representative images of Evans blue staining of the brain tissues. (**B**) Statistic analysis of Evans blue leakage (*n* = 7–8). (**C**) Statistic analysis of water content (*n* = 7–8). (**D**) Representative Western blots showing ZO-1, claudin-5, and VE-cadherin in the ischemic cortex. (**E**) Densitometry values for ZO-1, claudin-5, and VE-cadherin were normalized against those for the internal marker β-actin (*n* = 3). (**F**) The mRNA expression of MMP-2 and MMP-9 in ischemic brain tissues (*n* = 4). (**G**) Western blots of MMP-2 and MMP-9 in the cortex. (**H**) Densitometry value for MMP-9 was normalized against those for internal marker β-actin (*n* = 3). ^†^ *p* < 0.05, compared to Sham. ^‡^ *p* < 0.05, compared to Veh.

To examine the permeability of BBB after ischemic brain damage, we determined Evans blue extravasation, indicating the disruption of BBB. We found that pretreatment with MFSCE (10 mg/kg) significantly attenuated Evans blue extravasation in the ipsilateral hemisphere after ischemic brain injury compared with that in the vehicle group (*p* < 0.05; Figure 2A,B). To determine the effect of MFSCE on the post-ischemic occurrence of edema, we evaluated the brain water content at 24 h after ischemic brain injury. We observed that pretreatment with MFSCE significantly reduced the high water content in the ischemic brain compared with that in the vehicle group (*p* < 0.05; Figure 2C). To further investigate the role of MFSCE in the mechanism of BBB disruption, we measured the expression of the tight junction proteins ZO-1 and claudin-5 and that of the adherens junction protein VE-cadherin in the ischemic brain. Western blotting showed that the protein expression of ZO-1, claudin-5, and VE-cadherin was considerably higher in the MFSCE group than in the vehicle group (*p* < 0.05; Figure 2D,E). 

As shown in Figure 2F, pretreatment with MFSCE suppressed only the expression of MMP-9 mRNA (38.23% ± 10.83% of the vehicle group, *p* < 0.05), but not that of MMP-2 mRNA in ischemic cerebral tissues. We also confirmed the expression of MMP proteins in the ischemic cortex 24 h after ischemic brain injury. Correspondingly, we found that the protein level of MMP-9 was considerably decreased in the MFSCE-treated group compared with that in the vehicle group (56.03% ± 13.96% of the vehicle group, *p* < 0.05; Figure 2G,H).

### 3.3. MFSCE Prevented Ischemic Stroke-Induced Neuronal Cell Death and Activation of the NLRP3 Inflammasome

**Figure 3:**
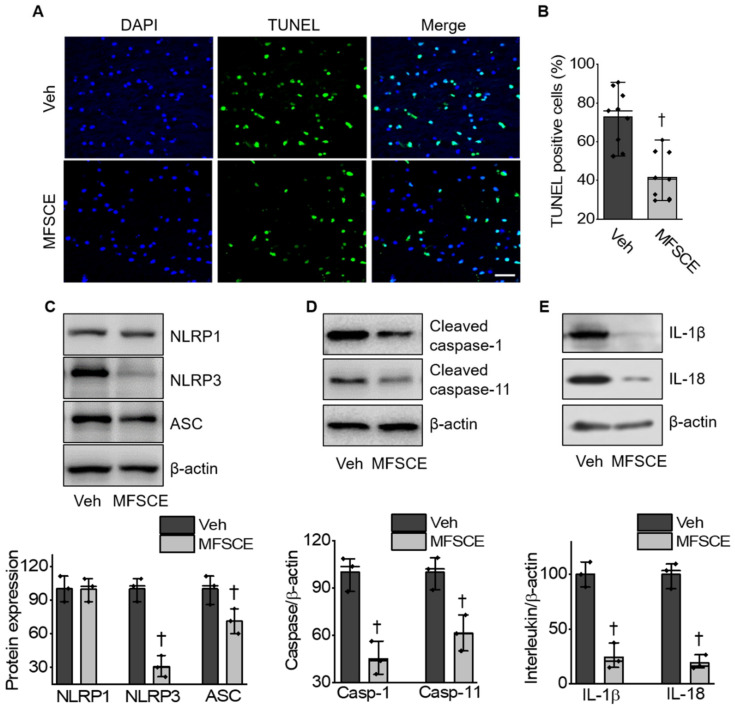
MFSCE suppressed neuron apoptosis by suppressing the inflammasome in ischemic brain injury. (**A**) Representative immunofluorescence for TUNEL (green) and DAPI (blue) staining. Scale bar represents 30 μm. (**B**) Statistic analysis of TUNEL (+)/DAPI (+) cells in the ischemic cortex (*n* = 9). (**C**–**E**) Pretreatment with MFSCE decreased the expression of NLRP3 and activity of the inflammasome in the ipsilateral side after ischemic stroke (*n* = 3). ^†^ *p* < 0.05, compared to Veh.

To determine whether MFSCE could reduce ischemia-induced neuronal cell death after ischemic brain injury, we subjected murine brain sections to TUNEL staining. MFSCE significantly decreased apoptotic neurons in the penumbra of ischemic mice compared with that in the vehicle group (41.69% ± 12.25% vs. 72.86% ± 14.39%, *p* < 0.05; Figure 3A,B). To further investigate whether MFSCE attenuates neuroinflammation after cerebral ischemia, we determined the expression of NLRP inflammasome-associated proteins, including NLRP1, NLRP3, ASC, CL-caspase-1, CL-caspase-11, IL-1β, and IL-18 in the ischemic cortex using Western blotting. We observed that the levels of NLRP3 and ASC were significantly decreased in the MFSCE group compared with those in the vehicle group (Figure 3C). In addition, MFSCE notably reduced the levels of cleaved caspase-1 and caspase-11 (Figure 3D), as well as those of mature IL-1β and IL-18 in the ischemic cortex compared with those in the vehicle group (Figure 3E).

### 3.4. MFSCE Attenuated TLR-4/NF-κB-Mediated Inflammatory Response in Mice with Ischemic Stroke

**Figure 4:**
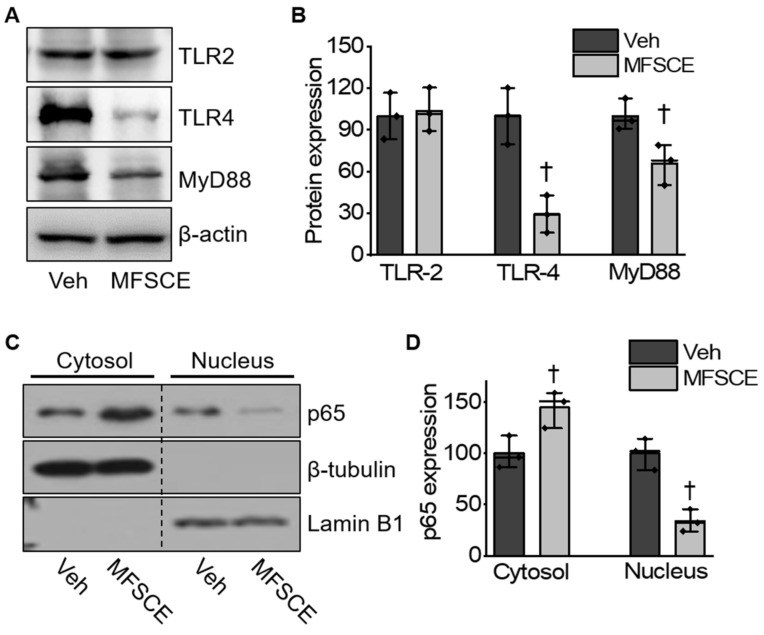
MFSCE exerted anti-inflammatory effect via the suppression of the TLR-4/p65 pathway in ischemic stroke. (**A**) MFSCE-induced reduction in the expression of TLR-4 and MyD88. (**B**) Densitometry values for TLR-2, TLR-4, and MyD88 were normalized against those for internal marker β-actin (*n* = 3). (**C**) MFSCE-stimulated inhibition of ischemic stroke-induced NF-κB nuclear translocation. (**D**) Densitometry value for p65 was normalized against those for internal marker Lamin B1 or β-tubulin (*n* = 3). ^†^ *p* < 0.05, compared to Veh.

To explore whether MFSCE protected against ischemia-induced neuronal cell injury through the TLR4/NF-κB pathway, we performed Western blotting. The expression of TLR-4, MyD88, and NF-κB was significantly upregulated in the vehicle group; however, these ischemic stroke-induced changes were markedly reversed by treatment with MFSCE (*p* < 0.05; Figure 4A–D). These results demonstrated that MFSCE could protect against cerebral ischemia by inhibiting the TLR4/NF-κB pathway. 

### 3.5. Preliminary Safety Test

**Figure 5:**
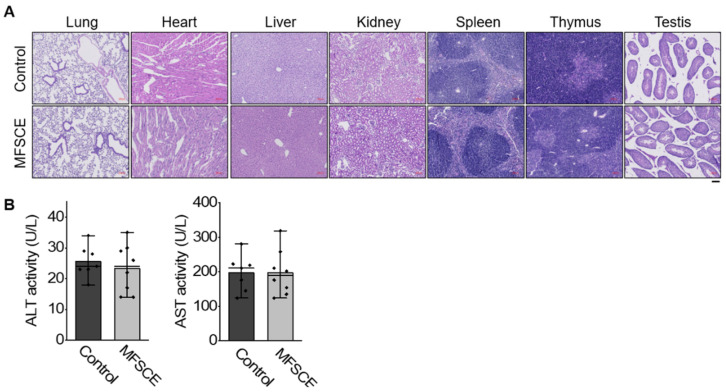
Safety profile of MFSCE. (**A**) Histochemical evaluation of H&E-stained lung, heart, liver, kidney, spleen, thymus, and testis sections of healthy C57BL/6 mice after intraperitoneal treatment with MFSCE at a dosage of 10 mg/kg for 5 days. Scale bar represents 100 μm. (**B**) Serum levels of ALT and AST after intraperitoneal treatment with MFSCE at a daily dosage of 10 mg/kg for 5 days (*n* = 7–8).

For initial safety evaluation, we tested the systemic toxicity of MFSCE in healthy C57BL/6 mice after intraperitoneal injection of MFSCE at a daily dosage of 10 mg/kg for 5 days. Compared with the control group, no deaths or serious bodyweight loss occurred in the two groups during the experimental period. We also noticed that major tissues, including the lung, heart, liver, kidney, spleen, thymus, and testis in mice of both groups had no obvious histopathological abnormalities or lesions (Figure 5A). Moreover, we did not detect any significant difference in the serum levels of AST and ALT between the MFSCE-treated and control group (Figure 5B), suggesting that intraperitoneal treatment with the current dosage of MFSCE did not cause systemic toxicity in mice.

## 4. Discussion

Our results showed that MFSCE of human adipose tissues reduced ischemic brain injury and BBB disruption in mice with ischemic stroke. The dosages of MFSCE (1, 3, and 10 mg/kg) used in this study were not the same as described in previous studies using MFSCE in mostly in vitro studies but were chosen from an arrangement that does not cause toxicity at the cellular level. Among the three doses of MFSCE tested, the highest dose was found to remarkably improve the stroke outcome. We demonstrated the beneficial effect of MFSCE on ischemic brain injury, which was accomplished by reducing the infarct volume and improving neurological and motor functions after focal cerebral ischemia. Regarding the permeability of BBB, we observed reduced Evans blue extravasation and water content in the brain of MFSCE-pretreated mice. In addition, pretreatment with MFSCE resulted in the upregulation of ZO-1, claudin-5, and VE-cadherin, and the downregulation of MMP-9 in the ischemic brain. Moreover, pretreatment with MFSCE suppressed NLRP3 inflammasome-mediated neuronal death, consistent with the inhibition of pro-inflammatory cytokines in the ischemic cortex through the inhibition of the TLR-4-mediated NF-κB pathway. However, MFSCE post-treatment did not affect functional recovery after cerebral ischemia. These findings suggest that intraperitoneally administered MFSCE may have a prophylactic rather than therapeutic effect for patients at high risk of stroke.

In recent years, stem cell-based approaches have emerged as attractive and promising new therapeutic options for ischemic brain injury. Animal studies have reported that the therapeutic effects of stem cells, including embryonic stem cells, inducible pluripotent stem cells, neural stem cells, and mesenchymal stem cells (MSCs), might involve cell replacement, neuroprotection, endogenous neurogenesis, angiogenesis, and modulation of inflammation and immune responses [24]. Among various types of stem cells, ADSCs have the following distinct advantages: high abundance, easy to obtain with minimal invasiveness, and cultured to a sufficient number for autologous transplantation without ethical issues [25]. In addition, ADSCs are known to secrete several growth factors such as nerve growth factor, brain-derived neurotrophic factor, glial cell line-derived neurotrophic factor [26,27], and vascular endothelial growth factor [28]. Therefore, the administration of ADSCs has been recently proposed as a new therapeutic option for the management and treatment of stroke. ADSC administration has beneficial effects on brain injury, by affecting cell death [16], apoptosis [16,25,29], inflammatory mediators [17,29], oxidative stress [25], cellular proliferation, neurogenesis, oligodendrogenesis, synaptogenesis, and angiogenesis [16]. Moreover, miR-31 from ADSC-derived extracellular vesicles (EVs) has been shown to downregulate the expression of TRAF6 and IRF5, leading to reduced ischemic stroke-induced neuronal damage [30]. Unlike EVs and MSCs, MFSCE does not include cellular contents such as membrane, DNA, and RNA. Therefore, MFSCE is not capable of self-division, thereby posing a very low risk when used as treatment compared with EVs and especially MSCs, which carry the risk of immunological rejection. In this respect, the dosages of MFSCE tested in this study and those of the MSCs used to treat stroke cannot be compared. Our study demonstrated that daily pretreatment of mice with MFSCE for 5 days before ischemic damage, considerably limited the size of the infarct brain and improved sensorimotor dysfunction after focal cerebral ischemia in mice (Figure 1).

The BBB, a highly selective semipermeable border of endothelial cells, limits and regulates the exchange of molecules, ions, and cells between the blood and central nervous system [11]. Stroke-induced dysfunction of cerebral capillaries results in a progressive alteration of the permeability of BBB; therefore, extravasations of high-molecular-weight compounds does not allow neurons to pass through the barrier compartments, along with water accumulation, vasogenic brain edema, and secondary ischemic injuries [31]. Thus, preserving the integrity of BBB during ischemia is an important therapeutic approach that requires further exploration. The tight and adherens junctions from the junctional complexes that make up the BBB comprise a complex network of transmembrane and cytosolic proteins, allowing them to seal and mediate the gate function of the BBB [32]. The tight junctions are degraded by MMPs, which are the predominant proteases involved in the disruption of the BBB following ischemic stroke [33]. Interestingly, MMPs are a family of zinc-dependent endopeptidases that have been identified as key mediators of BBB injury after cerebral ischemia. In particular, MMP-9 was found to be significantly increased in postmortem brain tissues obtained from patients with ischemic stroke, and was shown to be mainly localized in infiltrating neutrophils, endothelial cells, and activated microglia [34]. The increased expression and activation of MMPs play a pivotal role in thrombolysis-mediated BBB leakage and edema, resulting in intracranial hemorrhage [8]. Thus, MMPs are considered targets of a new therapeutic strategy for ischemic stroke through the protection of BBB. In mice with intracerebral hemorrhages, ADSC transplantations alleviates brain edema by inhibiting inflammation and Aquaporin 4 (AQP4) protein expression [29]. In addition, bone marrow MSCs have the ability to maintain the integrity of the BBB by suppressing AQP4 upregulation after cerebral ischemia [35]. In our study, the ischemia-induced disruption of the BBB was confirmed using Evans blue dye, whose signal was significantly reduced after pretreatment with MFSCE. In addition, treatment with MFSCE was found to ameliorate the increased water content in and edema of the ipsilateral brain. We also observed that MFSCE enhanced the expression of tight and adherence junction proteins, whereas it decreased the expression of MMP-9, indicating its protective effects on the disruption of BBB and edema in cerebral ischemia (Figure 2). 

Inflammatory responses play a pivotal role in ischemia-induced nerve injury. The dysfunction of BBB can cause neuroinflammation and the release of inflammatory cytokines, thereby promoting BBB damage [36]. The NLRP3 inflammasome has been proposed to mediate inflammatory responses and cell death during ischemic stroke. The NLRP3 inflammasome activates caspase-1, which cleaves pro-IL-1β and IL-18 into their active forms that are released into the extracellular environment [13]. The activation of TLRs and the NF-κB signaling pathway might induce the activation of NLRP3 inflammasomes, IL-1β, and IL-18 under ischemic conditions [14]. Various innate immune cells contain TLRs. Within the brain, they are mainly located on glial cells including microglia, astrocytes, and oligodendrocytes. Microglia and astrocytes express a wide range of TLRs [37] and produce pro-inflammatory cytokines when TLRs conjugate with their corresponding ligands [38,39,40]. A previous study suggested that TLR-4 might play a more important role than other Toll-like receptors during the course of brain damage caused by ischemia/reperfusion [41]. The levels of TLR-4 mRNA increased in neurons after 1 h of cerebral ischemia, which was accompanied by a high level of multiple inflammatory cytokines [42]. Similarly, a previous study revealed that TLR-4 knockout mice had significantly smaller infarct volumes and better neurological functions than wild-type mice after ischemia- and reperfusion-induced brain injury [41]. As a typical transcription factor, NF-κB is known to be activated during ischemic stroke. TLR-4-mediated MyD88-dependent signaling is essential for NF-kB activation [43]. MyD88 recruitment to the Toll/IL-1 receptor (TIR) domain of TLR-4 results in the activation of NF-kB, which produces pro-inflammatory cytokines. Additionally, inhibition of the activation of cerebral NF-κB and inflammation responses could contribute to protecting the BBB and attenuate ischemia-induced brain damage. ADSC transplantation reduces the level of pro-inflammatory factors (e.g., tumor necrosis factor-α, interferon-γ, IL-6, IL-1β, and IL-12), but increases the level of anti-inflammatory factors (e.g., IL-10) [44]. Furthermore, ADSCs or ADSC-conditioned media significantly reduced microglia migration and phagocytosis, as well as the secretion of pro-inflammatory factors, by suppressing microglial inflammation [45]. Moreover, exosomes from ADSCs relieved neural injury by inhibiting NF-κB and the mitogen-activated protein kinase (MAPK) pathway [46]. In this study, we demonstrated that the ischemic stroke-induced activation of NLRP3 was significantly reduced by MFSCE, as so were the downstream targets of cleaved caspase-1 and caspase-11, mature IL-1β, and IL-18 (Figure 3). In addition, MFSCE significantly downregulated the expression of TLR-4, but not that of TLR-2, while inhibiting the activation of NF-κB in the ischemic brain (Figure 4). Therefore, we believe that the neuroprotective effects of MFSCE could be exerted by the suppression of the NLRP3 inflammasome through the inhibition of the TLR4/NF-κB pathway. 

A major limitation of this study was that we focused on ADSCs obtained from only one source (patient with obesity); this may have influenced the expression of various factors in the adipose tissue stem cells that we obtained. Future studies should make use of several sources of ADSCs (individuals of different ages, sex, or obesity grade) for comparisons. Ischemic stroke is ranked as one of the leading causes of death, and post-stroke neurological disability is the most important problem to cause handicap worldwide. Until now, there is a limited prophylactic treatment that could be effective against neuronal damage and functional recovery, their importance is being recognized. The general purpose of prophylactic treatment is to reduce the likelihood that an individual will become ill, disabled, or die. However, it cannot be implemented in a “one-size-fits-all” manner. MFSCE may possibly be used as a novel alternative to prophylactic treatment options for high-risk ischemic stroke including patients with high blood pressure, heart disease, diabetes, family history of stroke, or transient ischemic attack, that requires early detection and preventive treatment. Although we found and preliminarily verified MFSCE plays an important role in protective effects against ischemic brain injury in mice, studies should continue to optimize this therapy and address the limitations, including the ability to effectively target the central nervous system, and exert beneficial effects while simultaneously avoiding adverse consequences. To the effective application of prophylactic treatment of MFSCE, methods that combine optimal delivery, dosages, and tracking will be needed and should be explored in varying animal models of cerebral ischemia to provide sufficient clinical evidence. Moreover, considering the physiological and anatomical differences between animals and humans, as well as the ethical limitations of clinical studies, it is necessary to evaluate the safety and effectiveness of MFSCE in patients with high-risk ischemic stroke through high-quality, large-sample, and multicenter prospective studies.

In conclusion, our results demonstrated that MFSCE, which is a stem cell component lacking the cell membrane, could successfully improve neural function after ischemic damage by attenuating the disruption of BBB and brain edema. This improvement might be achieved via the downregulation of NLRP3 inflammasome-mediated neuronal death, consistent with the inhibition of pro-inflammatory cytokines in the ischemic cortex via the suppression of the TLR-4-mediated NF-κB pathway. Therefore, pretreatment with MFSCE is a potential non-cell-and nontoxic-based stem cell therapeutic strategy for the prevention of cerebral ischemic injury. 

## Data Availability

Not applicable.

## Supplementary Materials

The following supporting information can be downloaded at: https://www.mdpi.com/article/10.3390/life12040503/s1, Figure S1: Post-treatment with membrane-free stem cell extract (MFSCE) did not improve tissue and functional outcomes after cerebral ischemia. Figure S2: Expended, uncropped Western blot panels from Figure 2 (*n* = 3). Figure S3: Expended, uncropped Western blot panels from Figure 3 (*n* = 3). Figure S4: Expended, uncropped Western blot panels from Figure 4 (*n* = 3).

## References

[1] Feigin V.L., Norrving B., Mensah G.A. (2017). Global burden of stroke. Circ. Res..

[2] Liu S., Levine S.R., Winn H.R. (2010). Targeting ischemic penumbra: Part I-from pathophysiology to therapeutic strategy. J. Exp. Stroke Transl. Med..

[3] Xiang H., Zhang Q., Han Y., Yang L., Zhang Y., Liu Q., Zhang Z., Zhang L. (2021). Novel brain-targeting 3-n-butylphthalide prodrugs for ischemic stroke treatment. J. Control. Release.

[4] Schwamm L.H. (2012). Major advances across the spectrum of stroke care. Nat. Rev. Neurol..

[5] Manning N.W., Campbell B.C., Oxley T.J., Chapot R. (2014). Acute ischemic stroke: Time, penumbra, and reperfusion. Stroke.

[6] Pfefferkorn T., Rosenberg G.A. (2003). Closure of the blood-brain barrier by matrix metalloproteinase inhibition reduces rtPA-mediated mortality in cerebral ischemia with delayed reperfusion. Stroke.

[7] Wang X., Lee S.-R., Arai K., Lee S.-R., Tsuji K., Rebeck G.W., Lo E.H. (2003). Lipoprotein receptor–mediated induction of matrix metalloproteinase by tissue plasminogen activator. Nat. Med..

[8] Lapchak P.A., Chapman D.F., Zivin J.A. (2000). Metalloproteinase inhibition reduces thrombolytic (tissue plasminogen activator)–induced hemorrhage after thromboembolic stroke. Stroke.

[9] Mazumder M.K., Bhattacharya P., Borah A. (2014). Inhibition of matrix metalloproteinase-2 and 9 by Piroxicam confer neuroprotection in cerebral ischemia: An in silico evaluation of the hypothesis. Med. Hypotheses.

[10] McColl B.W., Rothwell N.J., Allan S.M. (2008). Systemic inflammation alters the kinetics of cerebrovascular tight junction disruption after experimental stroke in mice. J. Neurosci..

[11] Yang C., Hawkins K.E., Doré S., Candelario-Jalil E. (2019). Neuroinflammatory mechanisms of blood-brain barrier damage in ischemic stroke. Am. J. Physiol. -Cell Physiol..

[12] Martinon F., Burns K., Tschopp J. (2002). The inflammasome: A molecular platform triggering activation of inflammatory caspases and processing of proIL-β. Mol. Cell.

[13] Schroder K., Tschopp J. (2010). The inflammasomes. Cell.

[14] Gross O., Thomas C.J., Guarda G., Tschopp J. (2011). The inflammasome: An integrated view. Immunol. Rev..

[15] Tsuji W., Rubin J.P., Marra K.G. (2014). Adipose-derived stem cells: Implications in tissue regeneration. World J. Stem Cells.

[16] Gutiérrez-Fernández M., Rodríguez-Frutos B., Ramos-Cejudo J., Vallejo-Cremades M.T., Fuentes B., Cerdán S., Díez-Tejedor E. (2013). Effects of intravenous administration of allogenic bone marrow-and adipose tissue-derived mesenchymal stem cells on functional recovery and brain repair markers in experimental ischemic stroke. Stem Cell. Res. Ther..

[17] Chi L., Huang Y., Mao Y., Wu K., Zhang L., Nan G. (2018). Tail vein infusion of adipose-derived mesenchymal stem cell alleviated inflammatory response and improved blood brain barrier condition by suppressing endoplasmic reticulum stress in a middle cerebral artery occlusion rat model. Med. Sci. Monit. Int. Med. J. Exp. Clin. Res..

[18] Venkatarame Gowda Saralamma V., Vetrivel P., Kim S.M., Ha S.E., Lee H.J., Lee S.J., Kim Y.S., Pak J.E., Lee H.J., Heo J.D. (2019). Proteome Profiling of Membrane-Free Stem Cell Components by Nano-LS/MS Analysis and Its Anti-Inflammatory Activity. Evid. Based Complement. Alternat. Med..

[19] Lee J.-K., Park M.-S., Kim Y.-S., Moon K.-S., Joo S.-P., Kim T.-S., Kim J.-H., Kim S.-H. (2007). Photochemically induced cerebral ischemia in a mouse model. Surg. Neurol..

[20] Lin T.-N., He Y.Y., Wu G., Khan M., Hsu C.Y. (1993). Effect of brain edema on infarct volume in a focal cerebral ischemia model in rats. Stroke.

[21] Hattori K., Lee H., Hurn P.D., Crain B.J., Traystman R.J., DeVries A.C. (2000). Cognitive deficits after focal cerebral ischemia in mice. Stroke.

[22] Uyama O., Okamura N., Yanase M., Narita M., Kawabata K., Sugita M. (1988). Quantitative evaluation of vascular permeability in the gerbil brain after transient ischemia using Evans blue fluorescence. J. Cereb. Blood Flow Metab..

[23] Kamoun W.S., Ley C.D., Farrar C.T., Duyverman A.M., Lahdenranta J., Lacorre D.A., Batchelor T.T., di Tomaso E., Duda D.G., Munn L.L. (2009). Edema control by cediranib, a vascular endothelial growth factor receptor–targeted kinase inhibitor, prolongs survival despite persistent brain tumor growth in mice. J. Clin. Oncol..

[24] Hao L., Zou Z., Tian H., Zhang Y., Zhou H., Liu L. (2014). Stem cell-based therapies for ischemic stroke. BioMed Res. Int..

[25] Leu S., Lin Y.-C., Yuen C.-M., Yen C.-H., Kao Y.-H., Sun C.-K., Yip H.-K. (2010). Adipose-derived mesenchymal stem cells markedly attenuate brain infarct size and improve neurological function in rats. J. Transl. Med..

[26] Wei X., Du Z., Zhao L., Feng D., Wei G., He Y., Tan J., Lee W.H., Hampel H., Dodel R. (2009). IFATS collection: The conditioned media of adipose stromal cells protect against hypoxia-ischemia-induced brain damage in neonatal rats. Stem Cells.

[27] Kalbermatten D.F., Schaakxs D., Kingham P.J., Wiberg M. (2011). Neurotrophic activity of human adipose stem cells isolated from deep and superficial layers of abdominal fat. Cell Tissue Res..

[28] Rehman J., Traktuev D., Li J., Merfeld-Clauss S., Temm-Grove C.J., Bovenkerk J.E., Pell C.L., Johnstone B.H., Considine R.V., March K.L. (2004). Secretion of angiogenic and antiapoptotic factors by human adipose stromal cells. Circulation.

[29] Zhang Y., Deng H., Hu Y., Pan C., Wu G., Li Q., Tang Z. (2019). Adipose-derived mesenchymal stem cells stereotactic transplantation alleviate brain edema from intracerebral hemorrhage. J. Cell. Biochem..

[30] Lv H., Li J., Che Y. (2021). miR-31 from adipose stem cell-derived extracellular vesicles promotes recovery of neurological function after ischemic stroke by inhibiting TRAF6 and IRF5. Exp. Neurol..

[31] Shi Y., Zhang L., Pu H., Mao L., Hu X., Jiang X., Xu N., Stetler R.A., Zhang F., Liu X. (2016). Rapid endothelial cytoskeletal reorganization enables early blood–brain barrier disruption and long-term ischaemic reperfusion brain injury. Nat. Commun..

[32] Lo E.H., Singhal A.B., Torchilin V.P., Abbott N.J. (2001). Drug delivery to damaged brain. Brain Res. Rev..

[33] Dejonckheere E., Vandenbroucke R.E., Libert C. (2011). Matrix metalloproteinases as drug targets in ischemia/reperfusion injury. Drug Discov. Today.

[34] Rosell A., Cuadrado E., Ortega-Aznar A., Hernández-Guillamon M., Lo E.H., Montaner J. (2008). MMP-9–positive neutrophil infiltration is associated to blood–brain barrier breakdown and basal lamina type iv collagen degradation during hemorrhagic transformation after human ischemic stroke. Stroke.

[35] Tang G., Liu Y., Zhang Z., Lu Y., Wang Y., Huang J., Li Y., Chen X., Gu X., Wang Y. (2014). Mesenchymal stem cells maintain blood-brain barrier integrity by inhibiting aquaporin-4 upregulation after cerebral ischemia. Stem Cells.

[36] Obermeier B., Daneman R., Ransohoff R.M. (2013). Development, maintenance and disruption of the blood-brain barrier. Nat. Med..

[37] Jack C.S., Arbour N., Manusow J., Montgrain V., Blain M., McCrea E., Shapiro A., Antel J.P. (2005). TLR signaling tailors innate immune responses in human microglia and astrocytes. J. Immunol..

[38] Bsibsi M., Ravid R., Gveric D., van Noort J.M. (2002). Broad expression of Toll-like receptors in the human central nervous system. J. Neuropathol. Exp. Neurol..

[39] Olson J.K., Miller S.D. (2004). Microglia initiate central nervous system innate and adaptive immune responses through multiple TLRs. J. Immunol..

[40] Bowman C.C., Rasley A., Tranguch S.L., Marriott I. (2003). Cultured astrocytes express toll-like receptors for bacterial products. Glia.

[41] Hyakkoku K., Hamanaka J., Tsuruma K., Shimazawa M., Tanaka H., Uematsu S., Akira S., Inagaki N., Nagai H., Hara H. (2010). Toll-like receptor 4 (TLR4), but not TLR3 or TLR9, knock-out mice have neuroprotective effects against focal cerebral ischemia. Neuroscience.

[42] Tang S.-C., Arumugam T.V., Xu X., Cheng A., Mughal M.R., Jo D.G., Lathia J.D., Siler D.A., Chigurupati S., Ouyang X. (2007). Pivotal role for neuronal Toll-like receptors in ischemic brain injury and functional deficits. Proc. Natl. Acad. Sci. USA.

[43] Seki E., Tsutsui H., Iimuro Y., Naka T., Son G., Akira S., Kishimoto T., Nakanishi K., Fujimoto J. (2005). Contribution of Toll-like receptor/myeloid differentiation factor 88 signaling to murine liver regeneration. Hepatology.

[44] González M.A., Gonzalez–Rey E., Rico L., Büscher D., Delgado M. (2009). Adipose-derived mesenchymal stem cells alleviate experimental colitis by inhibiting inflammatory and autoimmune responses. Gastroenterology.

[45] Yan K., Bian J.-R., He L., Song B.-W., Dong L., He J.-W., Shen L.-H., Zhou X.-Z., Zhen Y. (2021). Adipose-derived Mesenchymal Stem Cells Protect Neurons by Inhibiting Microglial Inflammation.

[46] Feng N., Jia Y., Huang X. (2019). Exosomes from adipose-derived stem cells alleviate neural injury caused by microglia activation via suppressing NF-kB and MAPK pathway. J. Neuroimmunol..

