# Supplementary material for: Membrane-Free Stem Cell Extract Enhances Blood–Brain Barrier Integrity by Suppressing NF-κB-Mediated Activation of NLRP3 Inflammasome in Mice with Ischemic Stroke
Source: Life (Basel). 2022 Mar 29;12(4):503. doi: 10.3390/life12040503 (PMC9032759; doi:10.3390/life12040503)
Supplement: Supplementary file 1 [file life-12-00503-s001.zip › life-1525516-supplementary.pdf]

Supplementary Materials

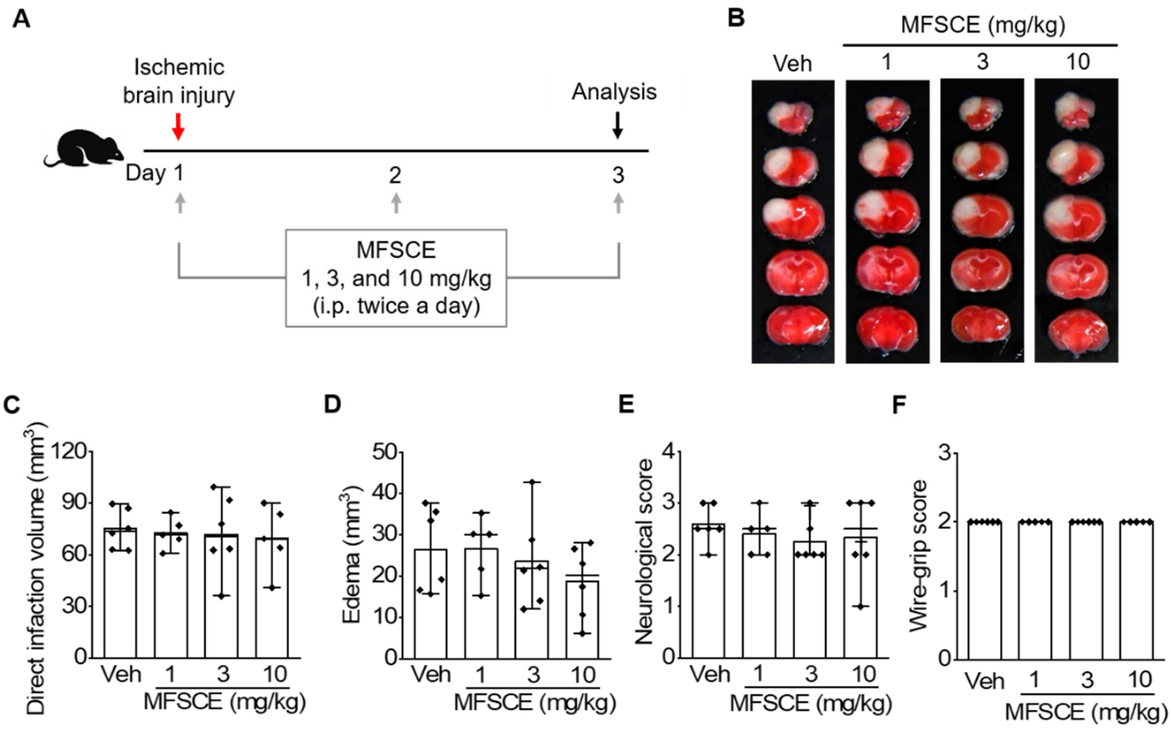

Figure S1. Post-treatment with membrane-free stem cell extract (MFSCE) did not improve tissue and functional outcomes after cerebral ischemia. (A) Scheme of the experimental protocol. Mice were administered a daily intraperitoneal (i.p.) injection of MFSCE (1, 3, and 10 mg/kg) in saline twice a day every 12 h for three days from 2 h after the induction of ischemic stroke. Focal cerebral ischemia was induced by photothrombosis of cortical microvessels. (B) Representative images of coronal brain sections stained with TTC in vehicle (Veh) and MFSCE-treated mice. (C) Direct infarct volume. (D) Edema. (E) Neurological score. (F) Wire-grip score (n = 5–6).

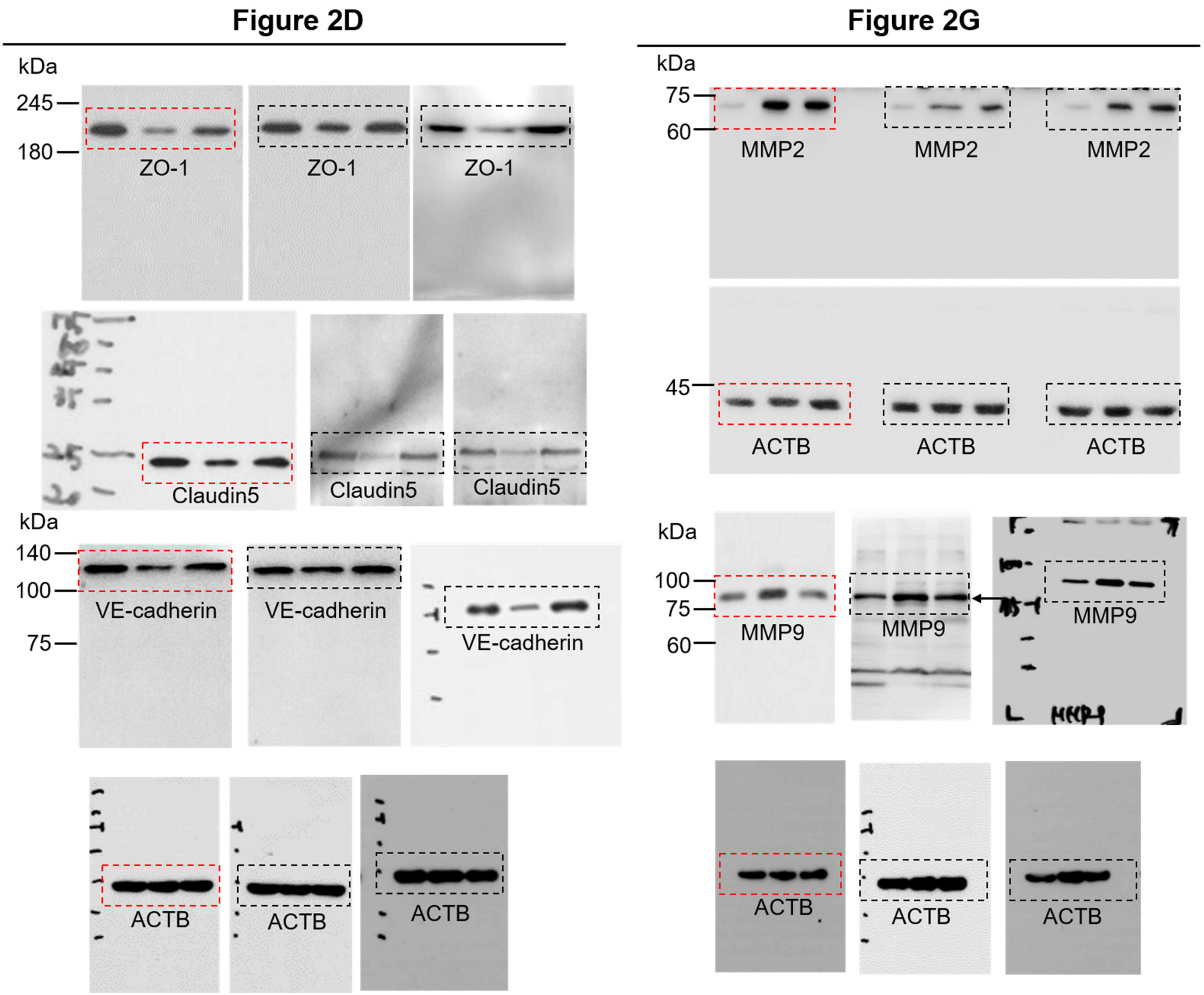

Figure S2. Expended, uncropped western blot panels from Figure 2 ($n = 3$).

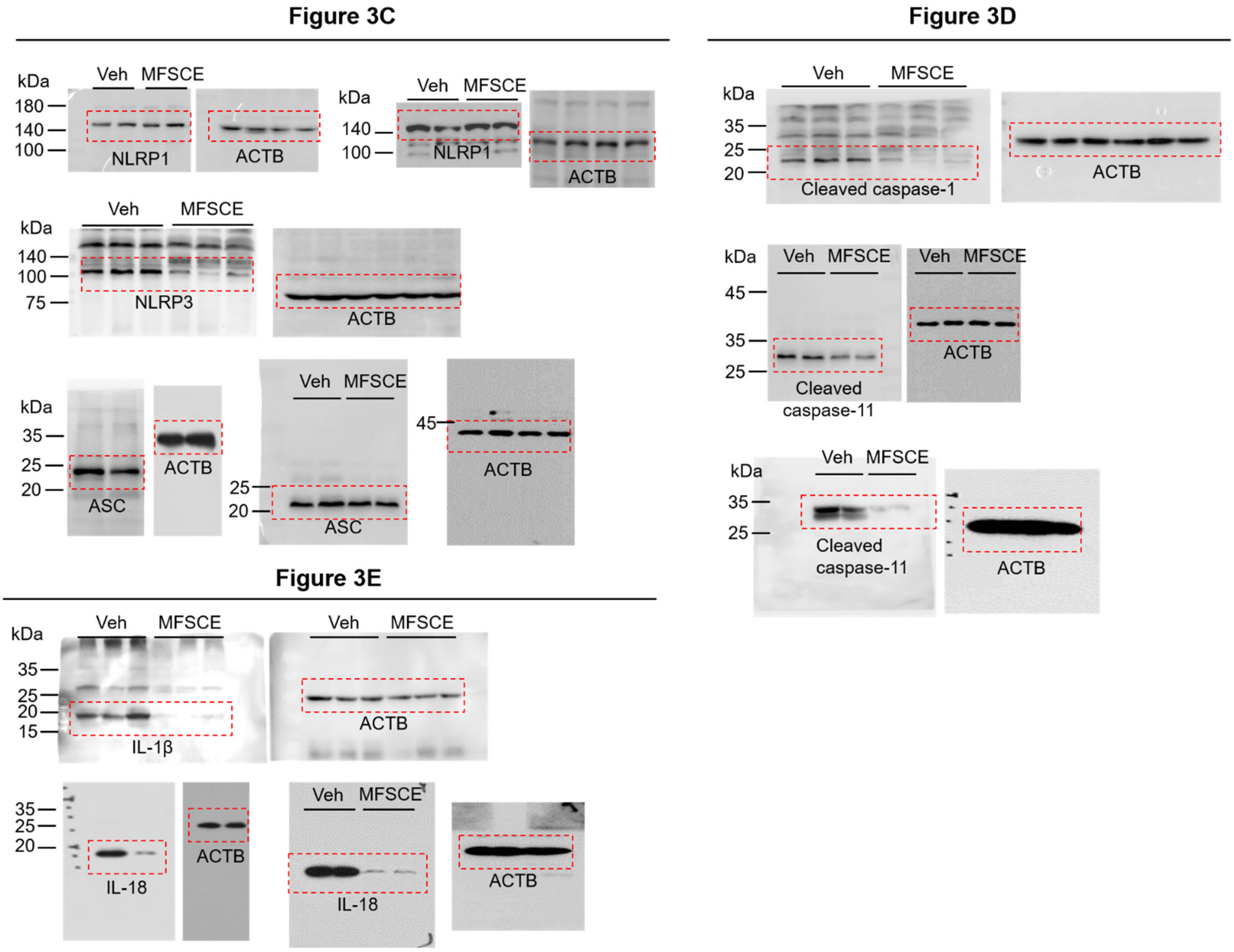

Figure S3. Expended, uncropped western blot panels from Figure 3 (n = 3).

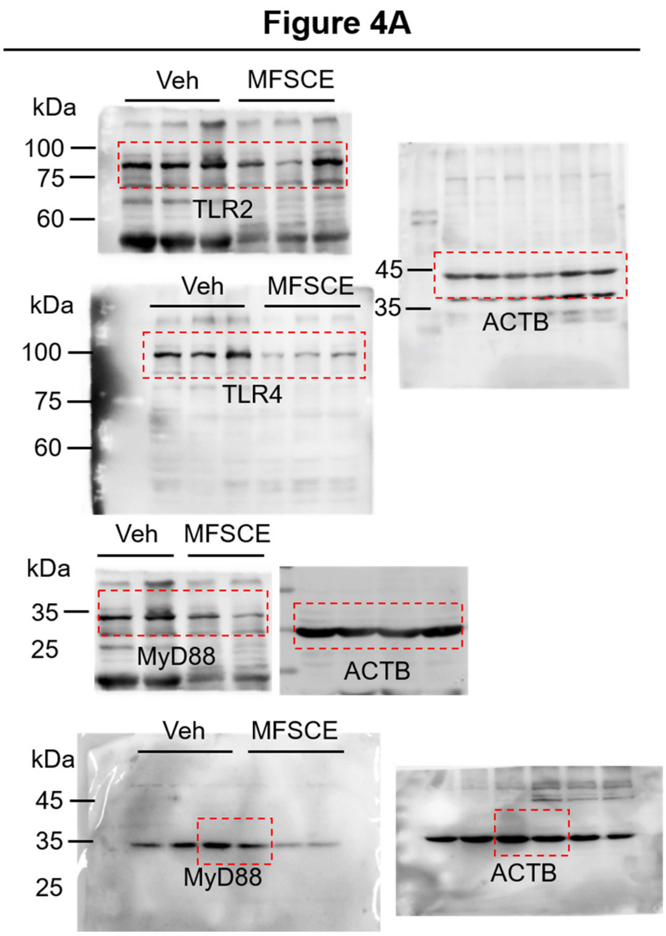

Figure S4. Expended, uncropped western blot panels from Figure 4 (n = 3).
